# Remarkable Case of Protracted Lactation

**Published:** 1847-04

**Authors:** I. P. Smith


4. *Remarkable Case of Protracted Lactation.* Mrs. P., aged 39 years October 28th, 1846, never had a sick day since her marriage December 9th, 1826, except the usual sickness consequent on parturition. During this period she has given birth to eight children, all of whom are now living and in perfect health. The order of their births is as follows:—Sept. 5th, 1827, female; Sept. 5th, 1829, female, March 28th, 1832; female; April 1st, 1834, female; November 11th, 1837, female; April 3d, 1841, male; April 17th, 1844, male; November 3d, 1846, female. Mrs. P.'s only brother and sister lived to adult age and both died of *tubercular phthisis.* Both parents also died of the same disease. She was married young, and at the time considered a remarkably slender girl, being subject to cough upon the slightest exposure. She has been constantly nursing for a period of nearly twenty years—never weaning one child till the birth of another compelled her to, for the convenience of the infant. More than once, when *in labor*, I have seen her child of the last birth at the breast.

From a solitary case of this kind, I would not draw a single inference; but should some of your numerous correspondents from the abundance of their experience, contribute for the Journal similar cases with a like favorable result, might we not infer, contrary to the generally received opinions of medical men, that protracted lactation, especially during pregnancy, possesses a prophylactic power, even when there exists a well-marked hereditary predisposition to pulmonary disease?—I. P. Smith, M. D., *in Ibid*,

